# Enhancing Nutritional Ingestive Behavior Microstructure Detection: Video Annotation and Passive Sensing Approaches

**DOI:** 10.3390/nu18101637

**Published:** 2026-05-21

**Authors:** Kathleen J. Melanson, Edison Thomaz, Nathan DeSalvo, Cody J. Arvonen, Adeleke J. Akinkurolere, Theodore A. Walls

**Affiliations:** 1 Department of Nutrition, University of Rhode Island, Kingston, RI 02881, USA; adeleke.akinkurolere@uri.edu; 2 Department of Electrical and Computer Engineering, The University of Texas at Austin, Austin, TX 78712, USA; ethomaz@utexas.edu (E.T.); codyarvonen@utexas.edu (C.J.A.); 3 Department of Psychology, University of Rhode Island, Kingston, RI 02881, USA; nathan.desalvo@uri.edu (N.D.); walls@uri.edu (T.A.W.)

**Keywords:** wearable technology, ingestive behavior microstructure, behavioral sensors, eating detection

## Abstract

Background/Objectives: Understanding the microstructure of ingestive behavior (IB) is critically important to the development and success of interventions to change eating rates and produce more optimal food energy intake outcomes. Detailed measurement of IB microstructure is needed to guide development of real-time sensing approaches that can support such interventions. This article summarizes novel measurement and inference strategies around both digital video and inertial motion sensors in a structured laboratory protocol. Methods: Digital video footage was annotated for chews and bites and analyzed with generalized additive models to assess differences in IB for each of four meal courses varying by food texture. Results: Significant differences were revealed in IB microstructure in the form of nonlinear patterns of annotated video footage and initial sensing tests, indicating an optimal sensor location over the jaw’s condyle bone. Conclusions: Findings of an intensive longitudinal multicourse full meal protocol reflect important differences in nonlinear trends of eating behavior for diverse texture foods. These differences inform further development of technology-aided measurement strategies, provide an experimental protocol for fieldwide IB inquiry, and reveal expected fundamental differences in ingestion rates. Further inquiry into the underlying causes of nonlinearities for high UPF foods, along with sensor measurements, is warranted.

## 1. Introduction

In order to address the global health burden of obesity and associated metabolic diseases, strategies for modifying eating behaviors must be implemented on multiple system levels [1]. Individual-level eating behaviors can be modified within and between meals to modulate food intake while enhancing homeostatic and hedonic satiety [2,3]. Typically, studies of ingestive behavior (IB) attend to the delivery of a bite, chewing and swallowing, from bite to bite with the goals of understanding their relationships with food intake [2,3,4] and body weight regulation [5]. Behavioral modification strategies then include tempering IB by altering patterns of ingestion through educational approaches, the use of technology, and/or manipulation of food properties [4]. Scholarship to date has mainly focused on testing these strategies within laboratory-based protocols under cross-sectional, cross-over or very short-term longitudinal designs. While the results have shown promise in these controlled settings, they lack external validity. Studies outside of laboratory settings have attended closely to the macrostructure of food intake (portion size, timing of meals, other eating experiences) and food types. Few studies have tested IB modification strategies in the real world due to lack of IB measurement capabilities outside of laboratory settings [6]. Ecologically valid, intensive longitudinal research on IB in people’s everyday lives is likely to be enhanced through the use of automated sensing technologies aimed at the nutritional ingestive behavior microstructure [7,8].

In this report, we describe two studies which comprise initial steps toward tracking IB microstructure at once using novel automated wearable technology that is ultimately intended for real-world use. In our first study, we describe our protocol and report results of analyses reflecting ingestion patterns revealed by a combined in-lab video monitoring and annotation approach. The results reflect preliminary data to support critical IB tracking. Our discussion considers IB patterns revealed by the video alone and considers the strengths and weaknesses of this approach versus possible benefits of sensor-based approaches. In our second study, we pursue development of a passive monitoring system for tracking ingestive behavior microstructure. We share pilot results reflecting hardware, initial testing of sensor locations, and anticipated machine learning approaches for use in future studies.

### Background

The combination of techniques such as taking small bites, thorough chewing, and increasing intra-bite pauses decreases overall eating rate and short-term food intake, and enhances satiation [1,9]. These techniques allow time for longer oral processing, esophageal passage, and prolonged gastrointestinal exposure, which have consistently been shown to improve satiety [2]. In contrast, prolonging meal duration without modifying bites, chews, or pauses fails to show consistently improved satiety [2], and modification of just one ingestive behavior can result in compensation via other ingestive behaviors, so that energy intake is not decreased [10]. Ingestive behaviors have been modified in adults with obesity through 5-week intensive interventions [1,11], as well as through an 8-week self-guided intervention [12]. In these two studies, lab-measured eating rate and energy intake were reduced in experimental groups but not in controls. These findings underscore the importance of modifying the ingestive behavior microstructure itself and not just watching the clock or putting down the utensil between bites. Effective slow eating combines smaller bites, lower bite rates, more chewing after each bite (chew-to-bite ratio) [9,13,14], extended oral processing, and longer pauses among bites and swallows [3,15,16]. Similarly, using universal eating monitor (UEM) data over 82 meals consumed by women with obesity, relationships were observed between ingestive behaviors and energy intake [11]. Multiple studies indicate that thorough chewing (mastication) [4,17,18] reduces energy intake [13,19,20,21], which may be mediated by enhanced satiety hormones [3,17,20,22]. Lower fasted- and fed-state hunger and lower lunch intake have been seen after chewing sugarless gum compared to non-chewing control testing, indicating a possible role of mastication in hunger reduction [23]. Modification of more than one IB at a time has been shown to be even more effective at reducing energy intake and enhancing satiety than modification of one IB alone [3,4,14,24]. For example, when people increase oral processing time and pause durations between bites, they consume fewer calories and show higher postprandial satiety hormones than when they engage in either of these alone, or neither [3]. In addition to enhanced satiety hormones, combining modified ingestive behaviors allows more time for satiety hormone signaling [3,4], along with improved insulin responsiveness [19], memory of what was eaten [25] and a higher thermic effect of meals [26]. Aspects of improved ingestive behaviors such as high chew-to-bite ratio (CBR) [4,14] can only be measured if both chews and bites are tracked; therefore, greater inquiry into the specific processes of ingestive behavior microstructure is sorely needed.

In order to give greater focus to this line of inquiry, we introduced an integrated conceptual model of the eating experience, which acknowledges macrostructure and food type, but centers on the key drive states of hunger, desire to eat, and satiety as a starting point [27]. These states drive the eating behavior process, and cause decision-making about eating to occur. Without any attention to ingestive behaviors, eating could proceed in an automatized way, which may be more or less facilitative of drive state reduction. This process is consistent with control concepts in which drive states are likely to be controllable through improved ingestive behaviors [28]. We hypothesize that consequences of long-term slowed ingestive behaviors include reduced overall energy intake and ultimately improved health outcomes associated with lower adiposity [2,4,5]. In order to test such hypotheses, advanced IB sensing methodologies are needed. Thus, the purpose of this work is to test a multimodal wearable device assembly that detects IB through bites and chews during a standardized four-course meal.

## 2. Materials and Methods

We conducted two laboratory studies in tandem to capture ingestive behavior microstructure in order to support the development of (a) a video and annotation approach, and (b) a prototype automated wearable technology. In single-visits, participants individually consumed a standardized test lunch while being discreetly (but not covertly) video-recorded. The test lunch included four separately served ad libitum courses: (1) chips and salsa, (2) baby carrots, (3) a homogeneous rice and beans dish, and (4) a churro, each course served along with water in a glass. The courses were chosen to reflect typical progressions of real-world meals for the purpose of external validity. Ingestive behaviors (bite counts, bite rate, chew count, chew-to-bite ratio, and pauses) were annotated from the video footage. In addition, a smaller group of participants were asked to wear facial sensors in three different locations on the face as well as a smartwatch, an assembly that we refer to as the Detection of Ingestive Behavior System (DIBS, version 1). The University of Rhode Island’s Institutional Review Board (IRB) approved the protocol (IRB2425-058), and all participants signed IRB-approved informed consent forms. Recruitment was accomplished through flyers, listservs, social media, newspapers, and classroom announcements in both Rhode Island and Texas. Eligibility criteria included ages 18–68 years and BMI 20–50 kg/m^2^, and those who expressed comfort being video recorded while eating. Exclusion criteria included allergies or intolerances to any of the food items, and difficulties chewing or swallowing. Participants were asked to abstain from alcohol consumption for 36 h and to fast (except water) for four hours before their planned lunchtime appointment to standardize hunger levels.

### 2.1. Procedures

For both studies, upon arrival to the lab for the individualized test visit, anthropometrics were measured: the participant’s height was measured in duplicate using a digital wall-mounted stadiometer (SECA 240, Hamburg, Germany), rounding to 0.1 cm, and the average height of the two measurements was recorded. Weight was measured in duplicate using a digital scale (SECA 700, Hamburg, Germany), rounding to 0.1 kg, and the average of the two measurements was recorded. Using these results, BMI was calculated as weight divided by height in meters squared.

Once these measures were complete, the participant sat in a separate, uniformly illuminated room within the lab. The video camera was situated on a tripod stand facing the participant’s jaw area, set to capture the person’s whole upper body, hands, and the food and beverage items on the table. The standardized test meal was served one course at a time, as shown in Table 1. All food items were weighed to 0.1 g on a kitchen food scale (Mettler Toledo ME204T; Columbus, OH) before and after participant consumption. For the second study, the DIBS tracked IB data (bite rate, chews, chew-to-bite ratio (CBR), and pauses).

### 2.2. Ingestive Behavior Microstructure Studies: Video Annotation and the DIBS Pilot

#### 2.2.1. Study 1. Video and Annotation Protocol Using ELAN

Meal sessions were video recorded for behavioral analysis. The camera was mounted on a tripod to ensure a stable view of the participant’s face and plate, with lighting verified before each session. Participants were seated in the testing room, informed about the meal protocol, and recorded throughout the meal. To minimize reactivity, the researcher remained out of view. Recordings were saved under standardized filenames including participant IDs. All videos were annotated in ELAN (version 6.9) using five synchronized tiers: bites, chewing episodes, and food type. Annotations were coded numerically, with “1” assigned to discrete events, total chew counts for chewing, and categorical codes for food type. Bites were defined as intervals from mouth opening to closure during food insertion, representing single ingestion events that initiate an oral processing sequence. Chewing episodes were annotated as continuous periods of mastication after food entry into the mouth and ended after ≥2 s without visible chewing. Within each chewing episode, chews were manually counted to capture variability in masticatory rhythm and intensity across foods. Food type was annotated as continuous intervals spanning each course and labeled according to the food served. Four annotators were trained to annotate the data according to each of the tier definitions. A codebook was provided to the annotators for training, outlining the precise definitions of each annotation type as described above.

To evaluate the inter-annotator agreement, each annotator annotated the data from the same two eating sessions, enabling an objective comparison before dividing the task and assigning each annotator their own video sessions to annotate. The annotations on the two eating sessions in common were updated by the annotators as the codebook was updated to resolve ambiguity until an adequate inter-annotator agreement score was achieved. A Fleiss’ Kappa of 0.82 and 0.85 were calculated for the bite and chewing episode tiers, respectively, across both eating sessions. Chew count values were assessed by comparing the total number of chews identified across an entire session by each annotator. The average percent difference in chew count between each annotator pair was 7.63%.

Together, these annotations provided a comprehensive description of ingestive behavior microstructure, linking oral processing phases (biting, chewing, drinking) with corresponding motor behaviors and food types. This tiered approach enabled the temporal alignment of ingestive actions, facilitating high-resolution behavioral analyses consistent with best-practice standards for the observational study of eating behavior.

#### 2.2.2. Statistical Analysis

Analyses were conducted in R (version 4.5.1) using the mgcv package [29] to fit generalized additive models (GAMs). The package implements automatic smoothness estimation and provides the gam() function used in these analyses. The underlying statistical framework is described in [30]. Data were structured at the level of session × food type × time bin, where ingestive behaviors were aggregated into 1 min intervals. Time was defined as minutes from the start of each food-specific eating block (i.e., the first bite for that food within a session).

For each time bin, bite rate and chew rate were calculated as the total number of bites or chews observed within that minute. The chew-to-bite ratio was computed as the number of chews divided by the number of bites within each time bin, excluding bins with zero bites. These derived variables represent rate-based measures rather than raw counts.

GAMs were selected to model nonlinear changes in these outcomes over time. Models were estimated using a Gamma distribution with a log link to account for positively skewed outcomes. Each model included a parametric effect of food type and separate smooth functions of time for each food, allowing behavior trajectories to vary across foods. Models were estimated by pooling observations across participants, with the goal of characterizing average time-varying patterns rather than individual-specific effects. Note that GAM-based approaches have also been successfully applied in recent nutrition and oral-processing research to model chewing and texture-related time trajectories [31,32].

Parametric coefficients represent differences between food types on the log scale and can be exponentiated to yield relative differences (e.g., exp(0.21) = 1.23 indicates a 23% higher rate). Smooth terms capture whether each outcome changes nonlinearly over time within each food condition.

#### 2.2.3. Study 2. Nutritional Ingestive Behavior Level—Microstructure Prototype (DIBS) Pilot

Measurement of microstructural IB, especially bites and chews, has been limited by a lack of technology to detect these behaviors in real-world settings. Eating is a complex process characterized by sequences of actions that include feeding (i.e., bringing food to mouth), chewing, and swallowing. Researchers have hypothesized that the use of specialized sensors to capture each of these actions individually and combine them into an integrated system can lead to more robust and accurate sensing. Fontana et al. explored this research direction, leveraging three sensing modalities that wirelessly interface to a smartphone: a jaw motion sensor, a hand gesture sensor, and an accelerometer in a system called AIM [33]. In a 24 h study with 12 participants, the system was able to detect food intake with an average accuracy of 89.8%. The jaw motion sensor was attached by medical adhesive below the earlobe and used to capture characteristic motion of the jaw during eating. The Automatic Ingestion Monitor (AIM) relied on a specialized RF-based proximity sensor to capture hand gestures. This sensor required participants to wear not only a wrist-mounted sensor but also a wireless module attached to a lanyard and worn around the neck. The AIM prototype demonstrated jaw movement tracking as well but with two key limitations. First, the jawbone movement sensor, a piezoelectric film element, had to be physically connected to the rest of the system. This shortcoming resulted in wires running from the face down to a microcontroller located in another part of the body (e.g., a pocket). Second, the AIM did not operate in real time; in the evaluation of the system, data were collected by 12 participants over 24 h and analyzed offline. Pioneering the use of head-mounted computing (i.e., Google Glass), Merck and colleagues combined head and wrist motion sensors with audio (i.e., a custom earbud microphone) for eating recognition [34]. Their approach was evaluated with 12 participants (six in the lab and six in the field), obtaining an average weighted accuracy of 92%. A similar solution based on head instrumentation has also been pursued [35]. Bedri and colleagues adopted the same approach to sensing and performed a more extensive evaluation [36]. They outfitted an eyeglass frame with a myriad of sensors to detect jaw movement, swallowing sounds, hand-to-mouth gestures, and food images. Using OCOsense glasses, Armitage et al. tracked facial movements and claimed to be able to detect several eating and chewing behaviors in a controlled lab study with 47 adults [37]. The OCOsense glasses correctly detected 81% of eating and 84% of non-eating behaviors. Despite encouraging results observed in prior work, the eyeglass frame design requires a high degree of personalization and is limited to individuals who need or are willing to wear glasses. More recently, Liu demonstrated that bio-impedance changes associated with eating activities can be detected on the wrist as individuals hold utensils, put food in the mouth, etc. These activities result in impedance variation. The downside of this approach is the need for electrodes to be placed on the hands or wrists [38].

The DIBS bears most similarity to the AIM of Fontana and Sazonov [33], but with important differences and refinements. Supported by prior work in commodity sensing which embeds sensing in commercial products [39,40], we use an off-the-shelf smartwatch for detecting food intake gestures. Smartwatches are scalable, socially acceptable and can capture motion with improved precision thanks to on-board inertial sensors (i.e., accelerometer, gyroscope, magnetometer). For chewing detection, our system relies on a small, inertial-based and wireless facial sensor. By streaming data to a custom-built application on a smartphone, the facial sensor tracks chewing activity and measures chew-to-bite ratio in real time. Beyond the advanced detection approach, innovation is inherent to the overall approach of tracking combined NIBM, onboarding the nuanced scientific specifications of IB in direct collaboration with nutrition, psychomotor and engineering scholars.

## 3. Results

### 3.1. Participants

Participant recruitment was conducted through a comprehensive strategy that included targeted advertisements on university bulletin boards, internal university listservs, pertinent online platforms (e.g., social media groups, research participation websites), and community outreach initiatives (e.g., flyers at local community centers as well as health, behavioral health, and weight loss clinics). Prospective participants completed an online screening form to evaluate their eligibility according to the specified inclusion and exclusion criteria detailed below. This recruitment approach sought to acquire a representative cohort of 30 healthy adults within the specified age range. This sample size was not determined based on a formal power analysis, but instead conforms to interdisciplinary norms for early testing of movement detection, as reflected in previous research [33,39].

As shown in Table 1, thirty adults participated in study 1: 63.3% were females (*n* = 19) and 36.7% were males (*n* = 11). They self-identified their race and ethnicity as: 46.7% Hispanic/Latino (*n* = 14), 30% White (*n* = 9), 13.3% Black or African American (*n* = 4), 6.7% Mixed Race (*n* = 2), and 3.3% Asian or Pacific Islander (*n* = 1). Participants’ ages varied from 18 to 46 years, with a mean age of 27.1 ± 7.8 years. The mean BMI was 27.1 ± 4.8 kg/m^2^, with a range of 18.3–38.9, indicating representation throughout normal, overweight, and obese classifications. Self-reported eating rate distributions were as follows: 40.0% indicated a very fast or fast rate, 36.7% a medium rate, and 13.3% as slow or very slow [41].

### 3.2. Video Data

#### 3.2.1. Bites per Minute—Comparisons Between Foods

**Figure 1:**
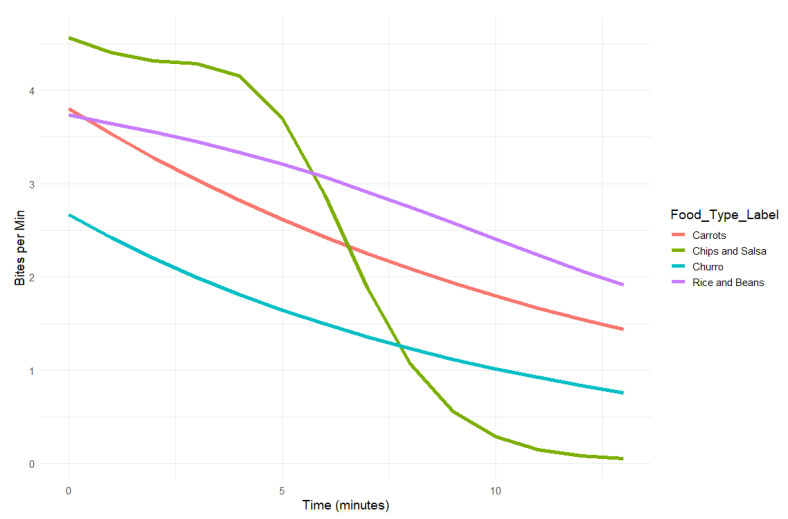
Predicted bite rate (bites per minute) over time by food, estimated using GAMs with a Gamma distribution and log link. All four foods showed statistically significant time-varying trends (Carrots, Chips and Salsa, Churro, and Rice and Beans; ps < 0.02). These lines reflect predicted changes in bite rate across the eating episode for each food.

We modeled bite rate (bites per minute) as a smooth, time-varying function across the courses using generalized additive models (GAMs) with a Gamma distribution and log link function (Figure 1). This approach accommodates skewed positive count data while capturing nonlinear changes over time, allowing the model to characterize how biting activity unfolds during an eating episode. The model included separate smooths for each course, treating Carrots as the reference category in the parametric component, with each having its own estimated bite rate curve. Carrots were chosen due to their use in the scientific literature as a basis for assessing habitual eating rate [42]. We refer to these smooths as time-varying trends. All courses had separate trends estimated.

The model explained 20.4% of the deviance in the data (adjusted R^2^ = 0.179), indicating a moderate fit. Time-varying trends were statistically significant for all four foods: Carrots (*p* < 0.001), Chips and Salsa (*p* = 0.005), Churro (*p* = 0.008), and Rice and Beans (*p* = 0.015), suggesting a meaningful change in bite rate over time for each.

Parametric (group-level) comparisons revealed significant differences between the foods in each course. The first course, Chips and Salsa, showed a significantly higher average bite rate than the second course, Carrots (b = 0.21, SE = 0.06, *p* = 0.001), corresponding to a 23% increase in predicted bite rate. Although the bite rate difference between Rice and Beans (third course) and Carrots (b = 0.08, SE = 0.06, *p* = 0.188) corresponded to an estimated 8% increase, this was not statistically significant. The last course, Churro, showed a significantly lower bite rate than Carrots (b = −0.40, SE = 0.07, *p* < 0.001), reflecting a 33% decrease. The model uses a log link, so exponentiated coefficients can be interpreted as relative rates; for example, exp(0.21) ≈ 1.23 indicates that the bite rate for the first course, Chips and Salsa, was about 1.23 times the bite rate for the second course, Carrots, providing an intuitive percent-based comparison across these courses. All smooth terms passed basis dimension checks (k-index ≈ 0.95, *p* > 0.20), and residual plots did not indicate major violations of model assumptions. These values likely reflect substantial within-person variability in bite behavior over time that is not fully captured by course order, food type, time and other meal-related factors; inclusion of additional predictors (e.g., bite size, palatability, or individual eating rate tendencies) may improve model fit in future work.

To quantify cumulative bite activity, AUC was computed for each course using the predicted bite curves. These reflect total predicted biting engagement across the eating episode but were not statistically tested. The AUC (total predicted bites) for each of the foods in the four courses were as follows: Carrots 31.6, Chips and Salsa 30.1, Rice and Beans 38.0, Churro 19.8 (courses 2, 1, 3, 4 respectively). These values reflect overall predicted biting engagement across each eating period.

#### 3.2.2. Chews per Minute—Comparisons Between Foods

**Figure 2:**
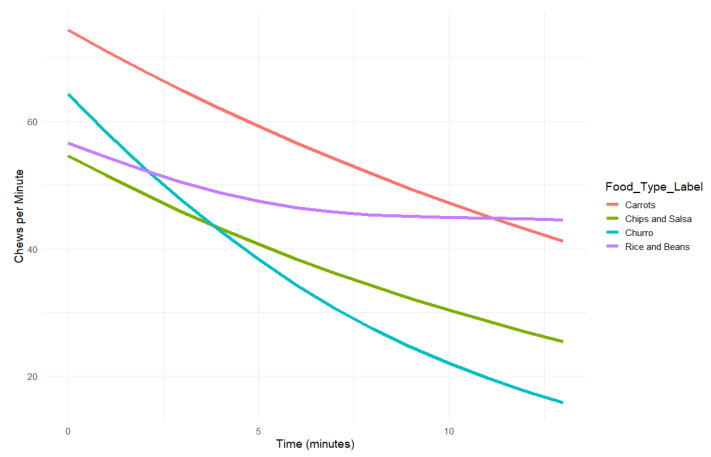
Predicted chew rate (chews per minute) over time for each food, modeled using generalized additive models (GAMs) with a Gamma distribution and log link. Each line reflects the estimated time-varying trend for a single food, based on model predictions. Trends were statistically significant for Carrots (*p* = 0.006), Chips and Salsa (*p* = 0.003), and Churro (*p* = 0.001). Rice and Beans did not show a significant time-varying trend (*p* = 0.086) and is included for descriptive purposes.

Because trends over meals were nonlinear, we modeled chew rate (chews per minute) as a smooth, time-varying function across the courses using generalized additive models (GAMs) with a Gamma distribution and log link function (Figure 2). This approach accommodates skewed positive count data while capturing nonlinear changes over time, allowing the model to flexibly represent how chewing intensity changes during the eating episode. The model included separate smooths for each food and treated Carrots as the reference category in the parametric component, meaning each food course had its own estimated chewing rate curve. We refer to these smooths as time-varying trends in the results. Bites and chews were estimated separately for each food course.

The model explained 9.2% of the deviance in the data (adjusted R^2^ = 0.14), indicating a modest fit (in Gamma models, deviance explained and adjusted R^2^ are calculated on different scales, so these values typically differ). Three of the four smooth terms were statistically significant (ps < 0.01), suggesting that chew rate varied meaningfully over time for most foods. Time-varying trends were statistically significant for Carrots (*p* = 0.006), Chips and Salsa (*p* = 0.003), and Churro (*p* = 0.001). Rice and Beans did not show a significant time effect (*p* = 0.086), suggesting relatively stable chewing over time for that food. Parametric (group-level) comparisons revealed significant differences between food types: Chips and Salsa showed a significantly lower average chew rate than Carrots (b = −0.34, SE = 0.05, *p* < 0.001), corresponding to a 29% decrease in predicted chewing rate. Rice and Beans showed a significantly lower chew rate than Carrots (b = −0.25, SE = 0.05, *p* < 0.001), corresponding to a 22% decrease. Churro also showed a significantly lower chew rate than Carrots (b = −0.27, SE = 0.06, *p* < 0.001), corresponding to a 23% decrease. These exponentiated coefficients provide relative rate interpretations: for example, a coefficient of exp(−0.34) ≈ 0.71 indicates that the chew rate for the first course, Chips and Salsa, was approximately 71% of that of the second course, Carrots. This provides an intuitive percent-based comparison across foods. All smooth terms passed basis dimension checks (k-index ≈ 0.77, *p* < 0.001), and residual plots did not indicate major violations of model assumptions, suggesting that the model adequately captured the main patterns in chewing behavior despite some individual outlier durations within course. These values likely reflect substantial within-person variability in chewing behavior over time that is not fully captured by course order, food type, and time alone; inclusion of additional predictors (e.g., bite size, palatability, or individual eating rate tendencies) may improve model fit in future work.

To quantify cumulative chewing activity, area under the curve (AUC) was computed for each food course using the predicted chew curves: Carrots 730, Chips and Salsa 497, Rice and Beans 621, Churro 456 (courses 2, 1, 3, 4 respectively). These were not tested statistically but help contextualize overall chewing demand across food courses. These values reflect overall predicted chewing engagement across the eating period, with higher AUC values indicating food courses that involved more total chewing.

#### 3.2.3. Chew-to-Bite Ratio—Comparisons Between Foods

We modeled the chew-to-bite ratio as a smooth, time-varying function across different food types using generalized additive models (GAMs) with a Gamma distribution and log link function. This approach accommodates frequently occurring positive skew in ratios and captures nonlinear changes in chewing behavior over the duration of each eating episode, allowing us to observe how chews per bite change across the eating episode. Carrots were used as the reference category in the parametric component of the model. Separate time-varying trends were estimated for each food.

The model explained 33.4% of the deviance in the data (adjusted R^2^ = 0.275), indicating a moderately strong fit across food types. Significant smooth terms were observed for Carrots and Chips and Salsa (ps < 0.05), suggesting that the chew-to-bite ratio changed meaningfully over time for these food courses. Rice and Beans and Churro did not show statistically significant smooth changes over time (ps > 0.13). Time-varying trends were significant for Carrots (*p* = 0.01) and Chips and Salsa (*p* = 0.03). Rice and Beans (*p* = 0.15) and Churro (*p* = 0.56) did not show significant nonlinear change.

Parametric (group-level) comparisons revealed the following differences in overall chew-to-bite ratio relative to Carrots: Chips and Salsa showed a significantly lower average chew-to-bite ratio than Carrots (b = −0.62, SE = 0.06, *p* < 0.001), corresponding to a 46% decrease in predicted chew-to-bite ratio (exp(−0.62) ≈ 0.54). Rice and Beans also had a significantly lower chew-to-bite ratio than Carrots (b = −0.30, SE = 0.06, *p* < 0.001), reflecting a 26% decrease (exp(−0.30) ≈ 0.74). Churro did not significantly differ from Carrots (b = 0.06, SE = 0.11, *p* = 0.562), indicating comparable chew-to-bite ratios between these two food courses. All smooth terms passed basis dimension checks (k-index ≈ 0.91, ps > 0.03), and residual plots indicated no major violations of model assumptions.

To quantify cumulative chewing behavior per bite, AUC values were calculated using the predicted chew-to-bite ratio curves across time for each food: Carrots 423, Chips and Salsa 219, Rice and Beans 271, Churro 211 (courses 2, 1, 3, 4 respectively). These values describe total modeled chewing per bite and were not tested for statistical differences. These results suggest that participants chewed substantially more per bite when eating Carrots, followed by Rice and Beans, while both Churro and Chips and Salsa resulted in lower overall chew-to-bite ratios.

### 3.3. The Detection of Ingestive Behavior System for Chewing and Inter-Bite Classification

Our two-device assembly consists of (1) a facial sensor for granular chewing and inter-bite detection, and (2) a smartwatch application for detecting intake gestures from arm, wrist and hand movements. We call the assembly the Detection of Ingestive Behavior System (DIBS). Both devices in the DIBS capture inertial signals and wirelessly transmit them via Bluetooth Low Energy (BLE) to a companion smartphone application. The application implements a pipeline that includes signal pre-processing, segmentation, feature extraction, machine learning classifiers and sensor fusion [43,44].

#### Facial Sensor Placement Study

**Figure 3:**
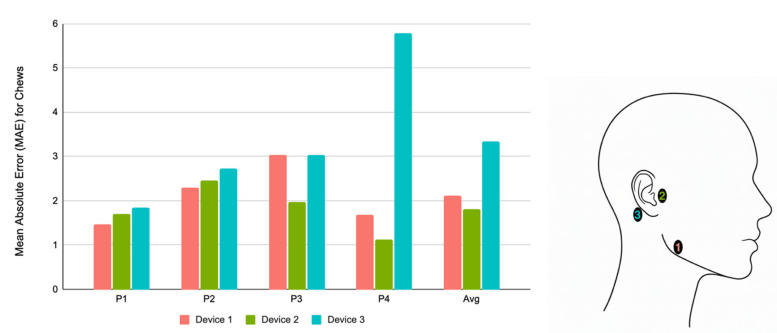
Relative performance of sensor locations. Device location 1 is on the mandible along the angle of the jaw, location 2 is on the mandibular condyle bone, and location 3 is behind the ear on the mastoid process.

To determine the optimal placement of the facial sensor, we conducted a pilot study with four participants (75% female, 25% male; 75% Asian, 25% Hispanic/Latino; average age 23.5 ± 4.1 years). These four participants followed the same protocol described for the 30 participants, except that they each wore three chew sensors simultaneously, one on each of the locations shown in Figure 3. Additionally, apple slices replaced carrots for this pilot study due to availability of materials at the time. Device location 1 is on the mandible along the angle of the jaw, location 2 is on the mandibular condyle bone, and location 3 is behind the ear on the mastoid process.

We trained a deep learning model to detect bites and the presence of chewing. The model architecture consisted of convolutional and recurrent neural network components, enabling it to learn temporal patterns in the data. We evaluated the ability of the model to generalize across data from unseen participants by using a leave-one-participant-out cross-validation scheme. This involved training the model on three participants and evaluating it with the fourth held out, and then repeating the process for each of the four participants. This evaluation was repeated for each of the three device locations individually. Additionally, we used a heuristic-based peak detection algorithm to detect and count the individual chews during a session, with each peak in the jaw motion data corresponding to an individual chew.

Overall, there was no significant difference between the device locations in the ability to detect bites and detect the presence of chewing. However, the devices do vary in performance when it comes to counting the number of chews. The plot below shows the performance of each device location for chew counting where the y axis is the MAE of chews for a participant across their whole session, calculated by averaging the absolute error of the predicted vs. the actual number of chews for each occurrence of continuous chewing (e.g., the chewing sequence between bites). On average, device location 2 performs the best across participants. While location 1 captured more overall motion as it experienced greater up and down displacement by following the jawbone directly, this added motion introduced more noise into the system. The lack of success for location 3, being behind the ear, is due to lack of jaw motion captured as this location is further from the jawbone. This is especially the case for participant 4, whose sensor on location 3 produced a particularly weak signal, resulting in a high error, highlighting a large variability and unreliability for this location across participants. Location 2, being placed directly on the mandibular condyle bone, can capture a strong signal from the jaw’s chewing motion while minimizing noise from extraneous jaw movements. This is the location we used for the sensor placement across all participants that were not a part of this sub-study.

## 4. Discussion

This work entails examination of ingestive behavior microstructure across a four-course meal with foods that vary in texture and consistency through two measurement studies. We began with video annotations and then conducted an initial assessment of a multimodal sensor detection approach for bites and chews. The first study revealed that significant, time-sensitive differences in IB could be detected by this methodology across the courses. The second study demonstrated baseline system functionality and relative facial sensor placement advantages.

### 4.1. Study 1

**Figure 4:**
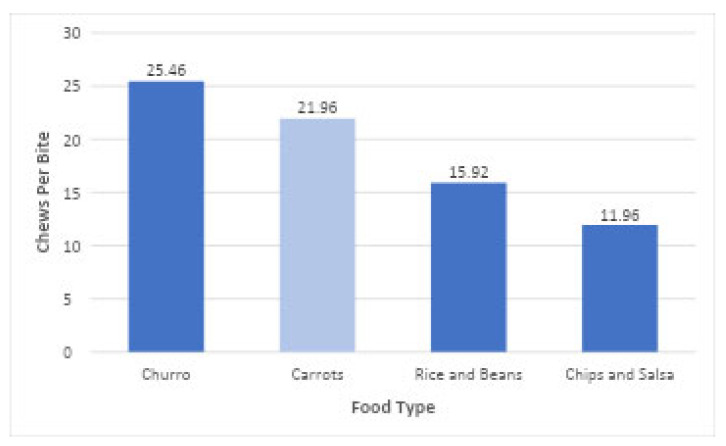
Chews and bites per minute by food types. Carrots are shown in light blue because they were selected as the reference group for the GAMs.

The four-course meal used in this protocol was designed to mimic a meal that may be consumed in real-world settings. The approaches tested were able to detect that, compared to the Carrot course, all other food courses elicited significantly fewer chews per minute. This second course differed in texture and has been used in other methodological work for testing differences in IB [42] (Figure 4). This aligns with work showing that texture and density strongly shape chewing effort: fibrous or crunchy foods demand significantly more chewing, while softer, moister foods can be prepared for swallowing with less mastication [45]. Its order within the earlier half of the meal may have influenced IB as well. All four food types showed overall reduction in chews per minute within their respective courses, which may reflect slowing of eating rates [46]. While slowing IB over the duration of a meal is typical in most humans [47], this repeated pattern within each of four courses during a meal has received less research attention. It possibly reflects sensory-specific satiation within each course of the meal [48]. This should be examined in future work. The third of the four courses, Rice and Beans, showed the flattest slope in the reduction in chews per minute. While it may be indicative of the lower mastication requirement of soft, moister foods, it may also be due to course order, palatability, bite size, or other factors. The first course, Chips and Salsa, elicited faster bite rates than Carrots, while the last course, Churro, elicited slower bite rates (Figure 4), which may be related to satiation. Rice and Beans fell in between, with bite rates that did not differ significantly from Carrots. These patterns are consistent with previous work showing that a food’s geometric form may influence the pace of intake [49]. However, while findings are consistent with this previous work, course order may have influenced bite rates, as people tend to eat faster at the beginning of meals when they are hungrier, and slow their pace as they become satiated [50]. Chips and Salsa, which was the first course, showed significantly higher bite rates than Churro, which was the last course of the meal. Within each course, bite rates declined for all foods, with a notably steep drop within the Chips and Salsa course. Any interpretations of the findings are purely speculative interpretations at this point. Their purpose is to provide a thought basis for the types of hypotheses that could be addressed through the types of methodological development presented in this paper.

**Figure 5:**
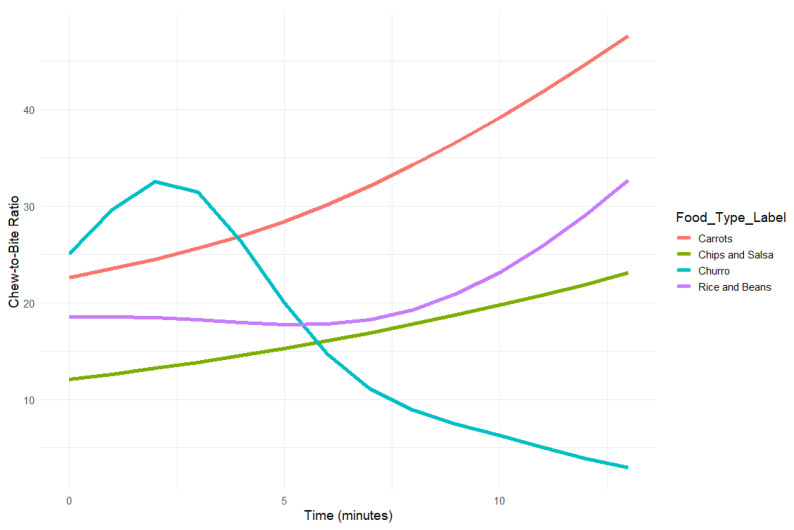
Predicted chew-to-bite ratio over time for each food, modeled using GAMs with a Gamma distribution and log link. Significant time-varying trends were observed for Carrots (*p* = 0.01) and Chips and Salsa (*p* = 0.03), while Rice and Beans (*p* = 0.15) and Churro (*p* = 0.56) did not show significant nonlinear change over time. Trend lines represent modeled change in chew-to-bite ratio across the eating episode.

Different chew-to-bite ratios were detected by the methodology during each of the food courses (Figure 5), with Churro and Carrots (fourth and second courses) showing higher chew-to-bite ratios than Rice and Beans and Chips and Salsa (third and first). The chew-to-bite ratio of the Churro did not differ significantly from Carrots. Extant scientific literature demonstrates differences in mastication across foods with differing structure, fibrosity, firmness, or moisture [46], as well as density [47] or geometry [49]. However, the time course was noticeably different between these foods, with Carrots showing an increase and Churro showing a decrease during the course. The increase in chew-to-bite ratio over each course for three of the four foods is of interest, as participants chewed more for each bite as the course proceeded, except for Churros, as shown in Figure 5. This may be related to changes in bite size with time, differences in the development of sensory-specific satiety, relative bite sizes and bite volumes, or subjective palatability of the foods [47]. These were not assessed in the current study, but they are hypotheses that can be addressed as this methodology progresses. The chew-to-bite ratio may be a useful metric for evaluating oral processing demands in different dietary contexts. It is open to scientific discussion regarding whether or under what circumstances it is best to use chew-to-bite ratio as the metric, or whether the ratio should be expressed as chews per weight or volume of each bite.

Because the first study revealed significant differences in chewing rate among the foods when subjected to a rigorous statistical analysis reflecting optimized calculations of rates, we suspect that our approach could lead to substantial changes in how these rates are parameterized and conceptualized. Specifically, these findings support the use of functional data analysis (FDA) and, specifically, generalized additive models (GAMs) for modeling behavioral eating dynamics. While GAMs enabled us to flexibly model nonlinear rate trajectories in this study, the broader methodological contribution lies in applying FDA to ingestive behavior. FDA offers a powerful framework for analyzing behavioral time series, especially those involving correlated, irregular, and non-Gaussian data structures, as is common in IB research. A growing number of studies across biomedicine, physiology, and public health have successfully applied FDA methods to model time-varying patterns in domains such as growth, motor behavior, and sensorimotor coordination [51]. Our findings add to this literature by illustrating how FDA can uncover meaningful patterns in biting, chewing, and their interaction over time, potentially offering more nuanced insights than traditional summary measures or linear models alone. In future work, these same GAM-based approaches can be applied directly to continuous raw sensor time series from the DIBS by modeling chewing-related signals as smooth functions of time, allowing for the extraction of time-varying patterns in ingestive behavior without reliance on manually annotated events. Our four-course meal protocol offers opportunities to examine IB dynamics within courses of different food types in addition to over the entirety of a meal. The model was designed to reflect the order of a typical real-world meal. Together with results from our sub-study, we hope that this will facilitate further development of our multi-modal IB sensing system in preparation for real-world settings, and to address hypotheses that were generated through this work.

### 4.2. Study 2

While detailed video annotation of chews and bites and use of a statistical approach that parameterizes rates very effectively are important advances, limitations to measurement and resource costs of the approach are of concern. Our initial results with the DIBS reflect system adequacy; therefore, we have moved to the initial development of a sensor-based system for measuring IB microstructure. Based on our findings, we chose to favor the use of one facial location over two other trial ones. Our immediate next steps are to optimize the sensor-based system, compare its adequacy to the video annotation approach, and deploy functional data analysis strategies in resulting sensor data.

In the medium to long-term, a significant difficulty involves improving the DIBS so that it performs satisfactorily in naturalistic settings. In real-world environments, false positive rates with automated dietary monitoring technologies have proven to be too high for most intended applications. Another challenge is practicality. A truly successful approach is one that can be adopted at scale, in a form factor that is socially acceptable. Many dietary monitoring sensing solutions featured in prior work showed encouraging performance but demanded the use and placement of sensors on the body in ways that would not be tolerable, such as large neck collars for swallowing detection [42,52,53] and microphones inside the ear canal to detect chewing [54]. Our aim is to further advance the DIBS to sidestep these limitations.

## 5. Conclusions

This work advances measurement tools and methods for automating the real-time characterization of ingestive behavior (IB) microstructure, addressing a critical need for multimodal sensing in real-world settings. IB microstructure is a key determinant of appetite and food intake, associated with broader eating behaviors, body weight, and health outcomes [27]. Even in the presence of otherwise healthy dietary practices, problematic IB patterns are associated with significantly higher food intake [4] and BMI [55], underscoring the importance of incorporating IB microstructure into comprehensive weight management strategies. The DIBS, the early-stage wearable system we developed, has the potential to delineate IB microstructure elements in context, enabling an ecologically valid characterization of actionable intervention points. For example, it has the potential to identify specific times of day, contextual factors, and eating patterns associated with risky IB, such as loss-of-control eating. Such capabilities could support the preliminary development of just-in-time interventions deployed across a range of strategies to promote healthier eating behaviors for the prevention and treatment of obesity and other health-related conditions.

Limitations and Next Steps. Despite these promising findings, this work has important limitations. In particular, the sample size is relatively small, as is typical for detection demonstrations, and the DIBS was evaluated only in a controlled laboratory setting, limiting external validity. This is particularly the case for our small second study of sensor location, in which one participant notably reflected much lower detection (cf. MAE, participant 4). Further, because foods were presented in a fixed sequence, food type is confounded with course order; therefore, observed differences may reflect both food characteristics and progression-related factors such as changes in hunger, satiation, or pacing across the meal. Future study designs will test other types of meals. Some individual outlier trends within food courses could have influenced the GAMs despite sufficient model fit, which is also something to be considered in further research and analyses. In general, we need expanded validation studies to ensure generalizability of both the systems in the lab and in field settings. Nevertheless, this work provides baseline engineering, inferential, and nutrition science contributions that lay an important foundation for leveraging practical wearable systems in IB measurement and nutritional health intervention research. Our prospective work will involve more robust testing of the sensor-based system against various sources of ground truth, further exploration of video-based detection of eating, analysis of intraindividual patterns of within meal eating, and efforts to explain more variance in bites and chews via exploration of the influence of covariates on IB patterns, utilizing larger sample sizes.

## Figures and Tables

**Table 1:** Demographic Characteristics of Participants (N = 30).

Characteristic	Category	*n*	%
Sex	Female	19	63.3%
	Male	11	36.7%
Race/Ethnicity	Hispanic/Latino	14	46.7%
	White	9	30.0%
	Black or African American	4	13.3%
	Mixed	2	6.7%
	Asian	1	3.3%
Age (years)	Mean (SD)	—	27.1 (7.8)
BMI (kg/m^2^) ^	Mean (SD)	—	27.1 (4.8)
	Range	—	18.3–38.9
Eating Rate *	Very Slow	1	3.3%
	Slow	3	10.0%
	Medium	11	36.7%
	Fast	11	36.7%
	Very Fast	4	13.3%

^ Body Mass Index; * Self-reported.

## Data Availability

Data supporting the findings of this study are not publicly available due to privacy and ethical restrictions involving identifiable video recordings.

## Abbreviations

The following abbreviations are used in this manuscript:

IB	Ingestive Behavior
UEM	Universal Eating Monitor
CBR	Chew-To-Bite Ratio
DIBS	Detection of Ingestive Behavior System
IRB	Institutional Review Board
BMI	Body Mass Index
GAMs	Generalized Additive Models
NIBM	Nutritional Ingestive Behavior Level—Microstructure
AIM	Automatic Ingestion Monitor
AUC	Area Under the Curve
BLE	Bluetooth Low Energy
MAE	Mean Absolute Error
FDA	Functional Data Analysis
